# Significance of TP53, CDKN2A, SMAD4 and KRAS in Pancreatic Cancer

**DOI:** 10.3390/cimb46040177

**Published:** 2024-03-23

**Authors:** Dimitrios Stefanoudakis, Maximos Frountzas, Dimitrios Schizas, Nikolaos V. Michalopoulos, Alexandra Drakaki, Konstantinos G. Toutouzas

**Affiliations:** 1 First Propaedeutic Department of Surgery, Hippocration General Hospital, School of Medicine, National and Kapodistrian University of Athens, 11527 Athens, Greece; stefanoudak@med.uoa.gr (D.S.); nmichal@med.uoa.gr (N.V.M.); 2 First Department of Surgery, Laikon General Hospital, School of Medicine, National and Kapodistrian University of Athens, 11527 Athens, Greece; dschizas@med.uoa.gr; 3 Division of Hematology and Oncology, David Geffen School of Medicine, University of California, Los Angeles, CA 90095, USA

**Keywords:** TP53, CDKN2A, SMAD4, KRAS, pancreatic cancer, PDAC, tumor suppressor genes, tumor markers, biomarkers, targeted therapy

## Abstract

The present review demonstrates the major tumor suppressor genes, including TP53, CDKN2A and SMAD4, associated with pancreatic cancer. Each gene’s role, prevalence and impact on tumor development and progression are analyzed, focusing on the intricate molecular landscape of pancreatic cancer. In addition, this review underscores the prognostic significance of specific mutations, such as loss of TP53, and explores some potential targeted therapies tailored to these molecular signatures. The findings highlight the importance of genomic analyses for risk assessment, early detection and the design of personalized treatment approaches in pancreatic cancer. Overall, this review provides a comprehensive analysis of the molecular intricacies of pancreatic tumors, paving the way for more effective and tailored therapeutic interventions.

## 1. Introduction

In 2023, there were 1,958,310 new cases of cancer and 609,820 cancer-related deaths in the United States. Among those, there were 64,050 new cases and 50,550 deaths related to pancreatic cancer (PaC). The absence of early detection and the limited effectiveness of the current chemotherapy are the main factors contributing to this high mortality rate. PaC is the third leading cause of cancer death among men and women, whereas mortality has increased slowly particularly in men [1]. The 2019 WHO classification of pancreatic tumors categorizes them into benign epithelial tumors (e.g., serous cystadenoma, intraductal papillary mucinous neoplasm), malignant epithelial tumors (e.g., duct adenocarcinoma, acinar cell carcinoma) and pancreatic neuroendocrine neoplasms (e.g., neuroendocrine tumors, functioning and nonfunctioning tumors) [2].

**Figure 1:**
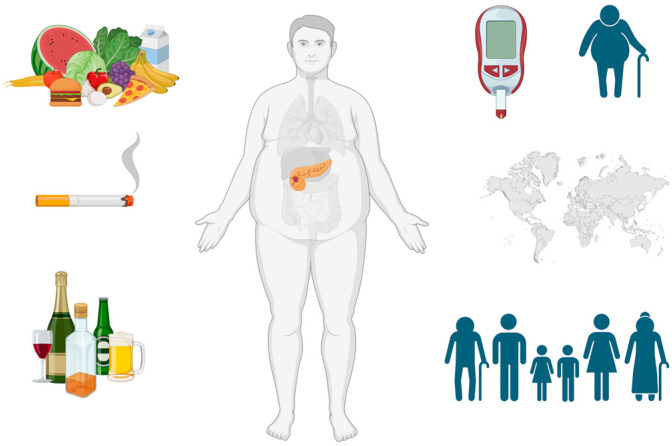
Modifiable factors include smoking, alcohol, obesity and dietary factors, where red meat increases risk, while vegetables and fruits offer protection. Non-modifiable risk factors comprise gender, age (with increased risk after 50), ethnicity, diabetes, family history, genetic factors, chronic infections and ABO blood group (non-O associated with higher risk). Created with BioRender.com (accessed on 27 February 2024) [13].

Pancreatic cancer commonly features mutations in key genes, such as KRAS, p16/CDKN2A, TP53 and SMAD4/DPC4 [3,4,5] (Table 1). Molecular profiling and biomarker identification like TMB, MSI and PD-L1 are needed in order to guide appropriate therapy [6]. In pancreatic cancer, frequent mutations occur in driver genes such as KRAS (88%), TP53 (77%), SMAD4 (29%), CDKN2A (18%) and TGFBR2 (7%) [7,8]. ATM loss, prevalent in 12.8% of PDAC cases, correlates with adverse clinicopathologic features and predicts decreased survival, particularly when combined with normal TP53 expression [9]. Somatic mutations, especially in KRAS, drive carcinogenesis and microRNAs, like miR-21, serve as prognostic biomarkers [10,11]. Lifestyle factors, notably smoking, contribute to elevated mortality, with a grim 5-year survival (2–9%), necessitating enhanced screening methods. Figure 1 summarizes the known modifiable and non-modifiable factors [12,13,14]. Although pancreatic ductal adenocarcinoma (PDAC) follows stepwise progression, surgical resection remains the primary curative option for a minority. Ongoing research explores neoadjuvant strategies and personalized treatments, facing challenges from intertumoral heterogeneity [15,16].

## 2. TP53

### 2.1. The Role of TP53 Mutations in Pancreatic Cancer

TP53 mutations, found in 50–90% of pancreatic ductal adenocarcinomas, significantly affect carcinogenesis, prognosis and treatment response [17,21,34,35]. The *p53* gene, located on chromosome 17, acts as a tumor suppressor by regulating cell division. Mutations in p53, which has a critical role in human cancer pathogenesis, lead to uncontrolled cell division. Understanding its impact on the tumor microenvironment and treatment response is crucial for developing effective therapeutic strategies for PDAC. TP53 mutations encompass about two-thirds missense and one-third truncating mutations, influencing mRNA degradation mechanisms [36]. These mutations, particularly gain-of-function (GOF) variants, alter the tumor microenvironment, promoting proliferation and chemotherapy resistance [34,36]. Cases with TP53 mutations often co-occur with KRAS mutations, suggesting early KRAS involvement in pancreatic carcinogenesis [36]. TP53 mutations affect the PDAC microenvironment, influencing immune responses, T-cell differentiation and interactions with cancer-associated fibroblasts (CAFs). TP53’s multifaceted role extends to metabolic regulation, shaping the hostile PDAC microenvironment by influencing metabolic reprogramming, autophagy and ferroptosis [34].

### 2.2. TP53 Mutations and Poor Prognosis

The TP53 mutational status-based genomic signature emerges as a critical factor for disease prognosis and therapeutic responses. Studies demonstrate its independent predictive value for overall survival and good prognostic estimate in pancreatic cancer patients. The accuracy of this genomic signature is underscored by its association with the immunophenotype of PDAC [21,22,23].

TP53 mutations, detected in 60–70% of cases, play a crucial role in suppressing malignant transformation, affecting carcinogenesis and prognosis [17,36]. TP53’s multifaceted roles include limiting preneoplastic lesion development, regulating their character and influencing the tumor microenvironment to restrain invasive cancer progression [17]. Remarkably, TP53 overexpression correlates with shorter overall survival (OS) [24]. Different molecular subtypes of pancreatic cancer exhibit diverse genetic alterations, with TP53 mutations contributing to disease progression [37]. The p53-Ptpn14-Yap axis emerges as a critical pathway in pancreatic cancer suppression, offering potential therapeutic avenues [18]. Furthermore, exosomal DNA analysis reveals high RAB27A, and TP53 expression independently associates with poor overall survival [25]. Genetic analysis indicates complex interactions among KRAS, CDKN2A, TP53 and SMAD4 alterations, influencing metastatic burden and survival [26].

TP53 mutations are prevalent in various pancreatic lesions, including 9.1% of intermediate-grade IPMNs, 17.8% of PanIN-2, 38.1% of high-grade IPMNs, 47.6% of PanIN-3 and 75% of invasive ductal adenocarcinomas. Notably, TP53 mutations were absent in pancreatic fluid samples from subjects with normal pancreas or chronic pancreatitis [27]. However, 5–10% of pancreatic cancers lack common mutations (KRAS, TP53, SMAD4, CDKN2A, CDKN2B), exhibiting diverse mutations, including therapeutic target alterations. Those tumors with unique molecular profiles lacking common mutations demonstrate improved overall survival, suggesting therapeutic relevance [28]. RAB27A and TP53 overexpressions serve as prognostic indicators and correlate with adverse clinicopathological features and independently indicate poor overall survival [25].

Genetically modified mice revealed distinct roles of mutant p53 in PDAC progression, demonstrating that coexisting KRAS mutation and mutant p53 (Trp53R172H) led to rapid PDAC onset with liver metastasis. Loss of p53 allowed the retention of KrasG12D-expressing cells, facilitating tumor formation and escape from KRAS-induced growth arrest. Mutant p53 accumulation promoted metastasis and was validated in human PDAC samples, where p53 accumulation correlated with lymph node metastasis. In vitro invasion assays confirmed the intrinsic role of mutant p53 in driving metastasis [19]. KRAS mutations, occurring early in pancreatic cancer progression, activate multiple signaling pathways, including Raf/mitogen-activated protein kinase and Akt/protein kinase B, influencing COX-2 transcription. Discrepancies in the mutational status of TP53 and CDKN2A/p16 among cell lines caution researchers about discrepancies between laboratory-specific and literature-reported mutational statuses [20]. TP53 plays a crucial role in suppressing malignant transformation in pancreatic cancer. Mutations occur in late-stage PanINs, indicating the p53’s role in inhibiting transformation into PDAC. In mouse models, intact p53 protects against pancreatic cancer progression, confirming its role in suppressing malignant transformation [17,29,36].

### 2.3. Diagnostic and Therapeutic Strategies Targeting TP53-Mutant Tumors

In pancreatic cancer, liquid biopsy using circulating cfDNA proves valuable for diagnosis and monitoring. A study successfully detected KRASG12D and TP53R273H mutations in exosomal DNA from PDAC patients, showcasing the potential role of liquid biopsy [30]. Moreover, specific miRNAs have been correlated with shorter survival. ctDNA analysis proved to be effective in early detection of disease progression and identification of actionable mutations, guiding therapeutic interventions [8,31]. Another study elucidates the ARF6–AMAP1 pathway’s role in promoting malignancy and immune evasion in PDAC, particularly influenced by KRAS and TP53 mutations. The pathway serves as a potential therapeutic target, emphasizing the significance of enhanced mRNA translation and protein geranylgeranylation in PDAC malignancy [32].

Introducing wild-type TP53 into cancer cells could alter sensitivity to treatments [18]. In particular, the TP53’s central role in PDAC progression explains the ongoing efforts to target mutant TP53 tumors, implicating a potential therapeutic option [33]. miRNA expression profiles in pancreatic cancer reveal prognostic significance while novel strategies involve exploiting the unique tumor microenvironment, such as using hyaluronidase to enhance drug delivery. Targeting KRAS, which plays a key role in PDAC pathogenesis, presents challenges, but inhibitors, like deltarasin, seem promising. Immunotherapy, successful in other cancers, faces hurdles in the immunosuppressive PDAC environment; however, vaccination strategies and immune checkpoint inhibitors are under investigation [37].

Overall, these findings highlight the potential role of liquid biopsy in combination with specific pathways influenced by KRAS and TP53 mutations, as well as the significance of TP53 in suppressing malignant transformation and shaping genomic signatures in pancreatic cancer.

## 3. CDKN2A

### 3.1. CDKN2A in Cell Cycle Regulation

Discovered in 1994, the *CDKN2A* gene, encoding the cell-cycle inhibitor p16, shows somatic mutations in various cancers [57]. CDKN2A, a critical tumor suppressor gene located on chromosome 9, encodes proteins such as p16(INK4A) and p14(ARF), playing pivotal roles in regulating diverse cancer-related processes. The encoded proteins exert their tumor-suppressive effects by inhibiting cell-cycle progression through binding and inhibiting cyclin-dependent kinases CDK4/6. This interaction maintains the retinoblastoma (Rb) protein in an active state, preventing G1 to S phase transition [109,110,111]. Additionally, CDKN2A promotes apoptosis and senescence and inhibits cancer-associated processes like cell-in-cell structure formation and anchorage-independent growth, while it modulates anti-tumor immunity by influencing immune-cell infiltration [38,58,109,111]. Dysregulation of CDKN2A, often observed through genetic and epigenetic alterations, is a common feature in various cancers, leading to uncontrolled cell proliferation and survival [58]. The *CDKN2A* gene, encoding tumor suppressor proteins like p16(INK4A) and p14(ARF), regulates cell growth and division by inhibiting cyclin-dependent kinases, preventing excessive proliferation and promoting processes like senescence and apoptosis [59,110]. Dysregulation of CDKN2A is associated with various cancers, including brain tumors, melanoma and lung cancer [110]. Located on chromosome 9, CDKN2A plays a crucial role in inhibiting cell proliferation and invasion across cancers [112]. Therefore, CDKN2A is an important gene in the regulation of cell growth and division, and its dysfunction can contribute to tumorigenesis.

### 3.2. CDKN2A Mutations in Pancreatic Cancer

CDKN2A mutations play a significant role in pancreatic tumors, with somatic mutations present in up to 95% of pancreatic tumors and a genetic predisposition observed in familial cases. The association with a higher risk of developing pancreatic cancer is evident, and families with CDKN2A germline mutations may exhibit a pancreatic cancer-melanoma syndrome [40,41,42]. In familial pancreatic cancer (FPC), CDKN2A mutations, along with those in BRCA2 and PALB2, were prevalent, particularly in FPC probands, highlighting their significance in hereditary pancreatic cancer [43]. Coexistent alterations in CDKN2A, KRAS, TP53 and SMAD4 were observed in pancreatic cancer, with frequent loss of tumor suppressors like CDKN2A, ARID1A, APC and ID3 [26,44]. In FPC families, CDKN2A mutations were identified in 21.4% of cases, and in patients with both pancreatic adenocarcinoma and melanoma, germline CDKN2A mutations (I49S and M53I) were found, with I49S showing impaired binding to CDK4 [45,46]. CDKN2A mutations were significantly associated with increased pancreatic cancer prevalence in families [47]. Germline CDKN2A mutations in familial melanoma-prone families showed diverse mutation types and variable pancreatic cancer distribution, suggesting genetic heterogeneity [48]. In non-Hispanic white pancreatic cancer patients, 0.6% had CDKN2A germline mutations, with higher rates in those with a family history, indicating its relevance in hereditary cases [40]. Inherited pathogenic variants in the *CDKN2A* gene contribute significantly to pancreatic cancer susceptibility, especially in FAMMM syndrome families. The risk of PDAC increases in individuals with pathogenic germline CDKN2A variants, even without a family history of melanoma. CDKN2A variants are identified in different populations, emphasizing variations in inherited risk. CDKN2A variant carriers face an increased risk not only for PDAC but also for melanoma and various other cancers. Despite the complexities of detecting CDKN2A variants of unknown significance (VUS), germline testing for CDKN2A is increasingly recommended for PDAC patients, offering opportunities for early detection through surveillance programs. Therapeutically, targeting the disrupted cell-cycle regulation by CDKN2A variants is explored through CDK4/6 inhibitors, showing promise in various cancers; although, the impact of CDKN2A status on treatment response requires further exploration [60].

Genome profiling of pancreatic adenocarcinoma revealed frequent homozygous deletions affecting CDKN2A/B and losses in TP53, PTEN and RB1. Amplifications were observed in GATA6 and MYC, emphasizing the complexity of genetic alterations in pancreatic cancer [49]. Notably, a study focusing on genetic alterations in pancreatic cancer highlighted the significance of CDKN2B deletion in tumorigenesis. Contrary to prior beliefs, the study found that CDKN2B, rather than CDKN2A, plays a crucial role in inducing pancreatic cancer. Deletion of CDKN2B, encoding p15INK4B, was identified as essential for tumorigenesis in a mouse model. This novel insight provides potential therapeutic strategies for pancreatic cancer treatment [39].

CDKN2A mutations and methylation play a crucial role in pancreatic cancer pathogenesis. Higher CDKN2A methylation is observed in pancreatic cancer patients, making it a potential diagnostic tool, particularly when analyzed in blood, pancreatic tissue and juice samples. The methylation is associated with different types of pancreatic cancers, including pancreatic ductal adenocarcinoma (PDAC) and pancreatic neuroendocrine tumors (PNET), correlating with shorter overall survival in both PNET and PDAC [50]. In individuals with the CDKN2A-p16-Leiden mutation, there is an increased risk of developing PDAC, with significantly better survival observed in cases with resected tumors. Surveillance strategies increase the probability of detecting PDAC at the resectable stage, potentially resulting in a 33.5% estimated long-term cure rate after surgery [51]. CDKN2A aberrations, including point mutations and deletions, are found in 25% of tumors. While CDKN2A aberrations alone do not significantly impact survival, concurrent mutations in both KRAS and CDKN2A are associated with the shortest survival, particularly in PDAC [52]. The accumulation of major driver alterations in pancreatic cancer, including CDKN2A, KRAS, p53 and SMAD4, is inversely associated with disease-free survival (DFS) and overall survival (OS), indicating a higher mortality risk with an increasing number of altered genes. This altered gene combination is specifically linked to liver metastasis [53].

### 3.3. Prognostic Value of CDKN2A and Therapeutic Strategies

The impact of CDKN2A mutations on overall survival in pancreatic cancer patients is still being investigated, with some studies suggesting a potential correlation with poorer prognosis in PDAC patients [54,55]. Importantly, higher CDKN2A expression is associated with improved prognosis. In particular, high CDKN2A expression is linked to activated immune cells, indicating its role in tumor immunity and its potential as a prognostic biomarker and therapeutic target [109]. Bioinformatics analysis of TCGA’s pancreatic adenocarcinoma data underscored the importance of CDKN2A inactivation in PDAC. Patients with CDKN2A mutations or deep deletions experienced poorer overall survival and primary therapy outcomes. CDKN2A-inactivated PDAC patients exhibited increased sensitivity to paclitaxel and SN-38, suggesting these as potential therapeutic options. The study also proposed paclitaxel as a potential treatment for CDKN2A-inactivated PDAC patients, as it mimicked the gene expression profile associated with CDKN2A restoration [54].

Therapeutically, PDAC with CDKN2A inactivation shows sensitivity to certain drugs like paclitaxel, indicating possible treatment strategies for tumors with these mutations. While targeted therapies may benefit patients with germline pathogenic CDKN2A variants and somatic loss, specific drugs are not detailed in the available information. Personalized treatment approaches, especially considering CDKN2A status, could enhance the efficacy of clinical trials for advanced pancreatic cancer. Overall, there is therapeutic potential in inhibiting the progression of pancreatic cancer by targeting CDKN2A mutations, but further research is essential to fully comprehend the effectiveness of these treatments and develop more precise and impactful therapeutic strategies [60,61]. The TAPUR Study, a phase II multi-basket clinical trial, investigated the anti-tumor activity of targeted agents in advanced cancers, including those with CDKN2A genomic alterations. In PDAC and gallbladder cancer (GBC) cohorts, single agent palbociclib, a Cyclin D Kinase 4/6 (CDK 4/6) inhibitor lacked clinical activity. However, whole exome sequencing and transcriptomic sequencing revealed a 23% rate of CDKN2A mutant status [56].

Understanding the genetic landscape of CDKN2A in pancreatic cancer provides valuable insights into risk assessment, surveillance strategies and potential targeted therapies for this challenging disease.

## 4. SMAD4

### 4.1. TGF-β Signaling and Tumor Suppression/Promotion

**Figure 2:**
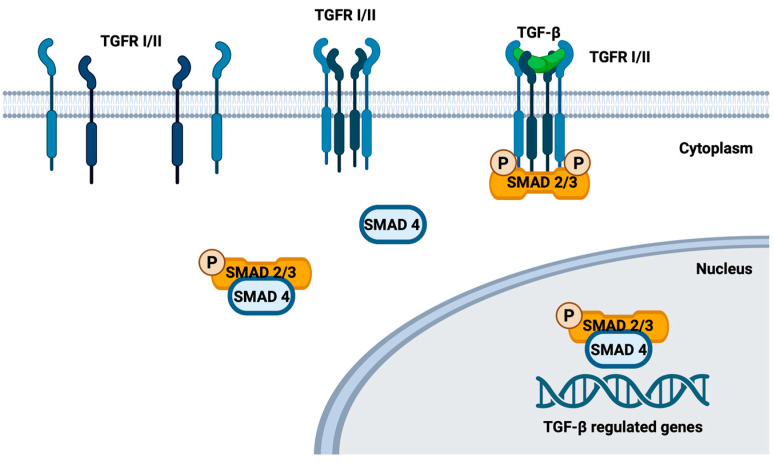
Schematic representation of the mode of action of TGF-β on cytoplasmic membrane receptors as well as the action of SMAD 4-SMAD 2/3 complex on intranuclear target genes: TGF-β binds to cytoplasmic membrane receptors, triggering downstream signaling cascades. One key pathway involves the formation of a SMAD4-SMAD2/3 complex, which translocates into the nucleus and regulates the expression of target genes, influencing various cellular processes. This mechanism illustrates how TGF-β signaling via SMAD proteins modulates gene expression and cellular responses. Created with BioRender.com (accessed on 27 February 2024).

SMAD4 is a crucial mediator in the Transforming Growth Factor Beta (TGF-β) signaling pathway, governing essential cellular processes like cell growth, differentiation, apoptosis and migration [81]. In this pathway, TGF-β binding to cell surface receptors triggers the activation of SMAD proteins, forming a complex with SMAD4. This complex translocates to the cell nucleus, regulating gene activity by binding to specific DNA regions [113] (Figure 2). Moreover, SMAD4 engages in a feedback loop by activating the transcription of its upstream receptors, thus maintaining pathway sensitivity [62]. This multifunctional protein also facilitates the activation of receptor-regulated SMADs (R-SMADs), including SMAD3 and SMAD1, highlighting its role in orchestrating downstream events [62,81]. Beyond the TGF-β pathway, SMAD4 participates in crosstalk with the Wnt signaling pathway by inducing the transcription of FZD4, a Wnt pathway receptor, emphasizing its broader regulatory impact on cellular functions [114].

In the context of tumorigenesis, SMAD4 is instrumental in inducing cell-cycle arrest and apoptosis, crucial mechanisms for controlling cell proliferation and eliminating damaged cells [81]. Furthermore, SMAD4 plays a pivotal role in the regulation of Epithelial-Mesenchymal Transition (EMT), a process with implications for cancer growth, wound healing and cancer metastasis [81]. Dysregulation of SMAD4 is associated with various aspects of cancer progression, encompassing autophagy, invasion and metastasis, underscoring its significance in cellular responses and disease [114]. SMAD4 also has a feedback regulatory function in the TGF-β signaling pathway. For instance, SMAD4 knockdown decreases TGFBR2 mRNA expression, whereas SMAD4 overexpression increases its expression [115].

In cancer, SMAD4 inactivation is frequent, notably in over half of PDAC and various other cancers. While usually SMAD4 loss alone does not initiate tumor formation, it promotes progression after cancer is initiated by other oncogenes like KRAS in PDAC and APC in colorectal cancer. However, in skin cancer, SMAD4 loss plays an initiating role by disrupting DNA damage response and repair [81].

### 4.2. SMAD4 Mutations and Pancreatic Cancer Behavior

The *DPC4* gene, located at 18q21.1, encodes Smad4 and is frequently altered in PDAC, with approximately 90% exhibiting loss of heterozygosity at chromosome 18q. A study investigated the role of AGR2, regulated by TGF-β and SMAD4, in PanIN progression. TGF-β1 down-regulated AGR2 in pancreatic cancer cell lines, and SMAD4 was identified as a key mediator in this regulation. AGR2, essential for MUC1 expression, interacts with MUC1 in the endoplasmic reticulum (ER), and its deficiency leads to reduced MUC1 expression in pancreatic lesions [116,117]. Smad4/DPC4, activated by TGF-β signaling, acts as a tumor suppressor gene. In pancreatic cancer, its inactivation is common, with immunohistochemistry effectively distinguishing benign from malignant states. A study involving 249 PDAC patients revealed that 43% lacked SMAD4 expression and 45% were positive. Genetic analysis showed concordance between SMAD4 expression and genetic status in 97% of cases. Patients with intact SMAD4 expression exhibited significantly improved prognosis, with a median survival of 19.2 months compared to 14.7 months for those lacking SMAD4 [66,82].

Analysis of 22 PDAC cell lines focused on genetic alterations in key cancer-related genes. Homozygous deletion of DPC4 was found in 32% of cases, exclusively present in cases with concurrent alterations in K-ras, p53 and p16. This suggests that DPC4 inactivation is a late-stage event in pancreatic carcinoma pathogenesis [67]. SMAD4 loss in PDAC has been associated with reduced lymphocyte infiltration, lower T-cell marker expression and decreased T-cell-mediated cytotoxicity. Patients with intact SMAD4 exhibited significantly better overall survival. Loss of SMAD4 impaired immune-related chemokine and cytokine synthesis, altered T-cell activation and reduced PD-L1 expression. Despite TGFβ signaling downregulating PD-L1 in vitro, SMAD4-intact tumors in vivo exhibited higher PD-L1 expression [68]. Another study investigated TGFB1-induced autophagy in PDAC progression, considering SMAD4 status. High LC3B expression, a marker of autophagy, correlated with TGFB1 and associated with pathways related to cell adhesion, migration and cancer. TGFB1-induced autophagy was more pronounced in SMAD4-negative cells. SMAD4 was found to be involved in autophagy induction by TGFB1 in SMAD4-positive cells. The dual roles of TGFB1-induced autophagy were revealed, inhibiting proliferation and promoting apoptosis in SMAD4-positive cells, while enhancing migration and the epithelial–mesenchymal transition. LC3B expression correlated with poor prognosis in SMAD4-negative PDAC patients, suggesting its potential as a prognostic marker [69].

### 4.3. Prognosis and Treatment Outcomes

SMAD4, a pivotal component of the TGF-β signaling pathway, plays a crucial role in regulating various biological processes and is integral to tumorigenesis. Its loss or mutation significantly contributes to cancer progression, making it a potential target for therapeutic interventions [81,113]. As aforementioned, in PDAC, SMAD4 mutations have profound implications, promoting tumor progression and metastasis, inducing resistance to radiotherapy and correlating with poor prognosis. These mutations impact stem-cell renewal, the epithelial–mesenchymal transition (EMT) and immune modulation, influencing treatment outcomes [63,64,68,70,83]. Specifically, SMAD4 mutations contribute to radiotherapy resistance through autophagy promotion, potentially serving as a biomarker for treatment efficacy [63]. While SMAD4 loss is associated with poor prognosis and shorter survival, its impact on chemotherapy response may vary, based on specific treatment regimens and disease stages [64,70,71].

The *DPC4* gene, encoding SMAD4, frequently experiences inactivation in PDAC, leading to enhanced metastasis and worse prognosis. Patients with DPC4-expressed cancers exhibit longer survival, emphasizing the significance of SMAD4 in treatment outcomes. Notably, SMAD4 loss is associated with distant metastases, highlighting its role in disease progression patterns [72,73,74,75,76,118]. Patients with an inactivated *DPC4* gene function have a higher risk of metastatic recurrence, with DPC4 inactivation being the most strongly correlated factor. Recurrence analysis indicates a significantly higher proportion of metastatic recurrences in the DPC4-inactivated group. DPC4-expressed cancers exhibit longer median overall survival (OS) and progression-free survival (PFS). Concurrent chemotherapy and local control show favorable outcomes, particularly in DPC4-expressed cancers [72]. In a cohort of 348 PDAC patients, DPC4/Smad4 expression loss was found in 53% of tumors. Univariate analysis links loss of DPC4/Smad4 to poor prognosis, but multivariate analysis reveals dependence on tumor size and lymph node involvement. In resected cases, loss of DPC4/Smad4 is associated with improved survival, suggesting it as a potential indicator for a beneficial response to resection [73]. Genetic alterations, including K-ras mutations, p53 and DPC4 expression, as well as c-erbB-2 overexpression, correlate with postoperative survival [74].

The loss of p16 and SMAD4/DPC4 immunolabeling has been associated with significantly shorter OS and DFS. Multivariate analysis identified the loss of SMAD4/DPC4 immunolabeling as an independent prognostic factor for overall and disease-free survival. The number of altered genes correlated with survival outcomes, with patients harboring three altered genes exhibiting significantly worse survival than those with one or two altered genes. Additionally, the loss of p16 immunolabeling was linked to distant metastases, emphasizing its association with disease progression patterns [75]. Among various gastrointestinal and extra-gastrointestinal carcinomas, the loss of SMAD4 staining is the most prominent in PDAC [76].

The majority (74%) of 69 patients in a phase II trial diagnosed with locally advanced (T4) pancreatic adenocarcinoma had unresectable tumors and were treated with cetuximab, gemcitabine and oxaliplatin, followed by chemoradiation with cetuximab. Treatment compliance was high, with 87% completing planned chemotherapy and chemoradiotherapy. Disease progression occurred in 69.6% of patients, with intact Smad4(Dpc4) expression associated with a local dominant pattern of progression, while Smad4(Dpc4) loss correlated with a distant dominant pattern [77]. In a univariate analysis, the loss of SMAD4 was significantly associated with poor OS in Asian patients, those with smaller sample sizes and those with a cutoff value of 0 [78]. Interestingly, mice with SMAD4 deficiency did not exhibit abnormalities in pancreas structure or physiology. However, when combined with the oncogenic KrasG12D mutation, Smad4 deletion dramatically accelerated the development of pancreatic tumors, leading to a significant reduction in survival [65].

The TGF-β/SMAD4 signaling pathway plays a pivotal role in pancreatic carcinogenesis, with TGF-β1 activating a SMAD4-dependent pathway crucial for regulating gene expression in normal pancreatic cells. However, in pancreatic adenocarcinoma, SMAD4-dependent TGF-β signaling is often inactivated, diminishing its tumor-suppressive effects. Over 50% of PDAC cases exhibit TGF-β pathway mutations, prominently involving Smad4, which is lost in 60–90% of cases. This loss facilitates tumor progression, metastasis and angiogenesis through Smad4-independent pathways, contributing to the complexity of PDAC. TGF-β also influences the tumor microenvironment, impacting immune responses and promoting fibrosis. In PDAC, elevated TGF-β levels are associated with increased metastasis and poorer prognosis, correlating with larger tumors, lymphatic and distant metastases, as well as advanced tumor stages, ultimately leading to reduced overall survival rates. Notably, Smad4 loss or inactivation is linked to adverse prognostic outcomes, with intact SMAD4 expression associated with significantly improved median and five-year survival rates. The frequent mutation of TP53 in conjunction with TGF-β/Smad4 alterations offers potential avenues for targeted interventions in pancreatic cancer [84]. Studies assessing SMAD4 expression’s prognostic significance in resected pancreatic cancer highlight its critical role. In one study, 59.8% of specimens were SMAD4−, correlating significantly with adverse clinicopathological parameters and adverse EMT status. Patients with SMAD4+ experienced significantly better disease-specific and disease-free survival compared to their SMAD4− counterparts. Multivariate analysis identified SMAD4− as the most prominent prognostic factor for PDAC, emphasizing its significance alongside other factors like elevated CA19-9 levels and metastatic characteristics [79].

Another study involving 237 patients identified SMAD4 loss and an activated Hedgehog (Shh) signaling pathway as predictors of poor prognosis. Patients with SMAD4 loss, high Gli1 and SMO expressions had significantly worse overall and recurrence-free survival. The integrated model combining SMAD4 status, Gli1 and SMO expressions demonstrated superior prognostic strength compared to individual variables, suggesting potential clinical utility. The study proposed the loss of SMAD4 in conjunction with an activated Shh pathway as a predictive factor for prognosis in PDAC, emphasizing the potential clinical significance of this molecular profiling in patient counseling and disease management [80]. In a study involving 95 PDAC cases, SMAD4 Y353C mutation, a novel mutation, was identified in 75.7% of carcinoma tissues, correlating significantly with malignant phenotypes. Functional analyses showed that this mutation resulted in lower SMAD4 expression in vitro. SMAD4 Y353C promoted epithelial–mesenchymal transition (EMT), increased cell migration and invasion, and altered E-cadherin and Vimentin expression. Despite associations with malignant phenotypes, SMAD4 Y353C did not affect the overall survival rate. This study suggests that SMAD4 Y353C may act as a tumor suppressor gene, emphasizing its potential as a therapeutic target and the need for further research to explore its mechanisms and implications for PDAC treatment [70].

Therapeutic approaches targeting SMAD4 mutations in pancreatic cancer seem promising, particularly in the realm of T cell-related therapy, indicating potential avenues for immunotherapy. Additionally, strategies inhibiting autophagy, a process promoted by SMAD4 mutations and implicated in radiotherapy resistance, could enhance the effectiveness of radiotherapy in SMAD4-mutant tumors. The TGF-β/SMAD4 pathway, disrupted by SMAD4 mutations, emerges as a potential therapeutic target for treating SMAD4-mutant tumors [85,119].

However, several therapeutic barriers exist. SMAD4 mutations can lead to resistance to radiotherapy through the promotion of autophagy, suggesting that SMAD4 status could serve as a molecular biomarker for PDAC but also posing a challenge for effective treatment. The impact of SMAD4 mutations varies among different tumor types, contributing differently to tumor initiation and progression. For instance, SMAD4 loss alone may not initiate tumor formation but can promote tumor progression initiated by other genes, such as KRAS activation in pancreatic ductal adenocarcinoma and APC inactivation in colorectal cancer. This variability presents challenges in developing effective, targeted therapies [64,81].

Moreover, TGF-β-mediated suppression of AGR2, partially mediated by SMAD4, implicates AGR2 as a potential molecular target for PDAC prevention and treatment. AGR2 deficiency delays PDAC initiation and progression in a mouse model, suggesting its integral role downstream of oncogenic KRAS. Additionally, a study exploring *SMAD4* gene mutation’s impact on pancreatic cancer response to radiotherapy reveals that SMAD4 depletion induces resistance to ionizing radiation. Knocking down SMAD4 in pancreatic cancer cells leads to increased radio-resistance, heightened DNA damage, genomic instability and decreased levels of key DNA double-strand break repair proteins [64,117].

## 5. KRAS

### 5.1. KRAS Mutations in Pancreatic Tumors

The prevalence of KRAS mutations in pancreatic tumors is striking, with mutations of the *KRAS* gene being present in 90–95% of pancreatic adenocarcinomas, making it the most frequently mutated gene in this type of cancer [97]. In particular, the KRAS isoform is mutated in 84% of all RAS-mutant cancers, with a near 100% mutation frequency in PDAC. This high prevalence is significant as it makes PDAC arguably the most RAS-addicted cancer, with substantial experimental evidence that mutant KRAS is essential for its growth [107]. The most common KRAS mutations in patients with PDAC are found in codons 12, 13, and 61, with G12D being the most common mutational substitution. Despite the high prevalence of KRAS mutations in pancreatic cancers, only a limited number of cases harbor an actionable point mutation, which poses a challenge for targeted therapies [120].

One KRAS (Kirsten rat sarcoma viral oncogene homolog) mutation is present in up to 25% of all human tumors, and this is one of the most frequently activated oncogenes. Recent research has demonstrated that the presence of the KRAS mutation may directly influence medical decisions in patients with cancer [121]. Oncogenic KRAS mutation plays a crucial role in the initiation and progression of PDAC by inducing reactive oxygen species (ROS) generation through metabolic changes. This excess ROS triggers key signaling pathways implicated in PDAC development [122]. In a study, pancreas-specific blockade of TGF-β signaling, combined with active KRAS expression, led to aggressive PDAC development with enhanced progression, metastatic potential, and invasion [86]. The stepwise progression from intraepithelial neoplastic lesions to adenocarcinoma in pancreatic cancer involves early events of oncogenic KRAS mutations. The interplay of KRAS and EGFR signaling pathways underscores the need for a multifaceted understanding of their dynamics for potential therapeutic interventions and personalized treatment strategies [108]. Importantly, a study conducted genomic analyses on pancreatic cancer, involving 456 tumors primarily of PDAC and its variants. The research identified 32 significantly mutated genes grouped into 10 molecular mechanisms, including KRAS mutations in 92% of cases. A copy number analysis revealed recurrent gains and losses, implicating genes like MET and CDKN2A [87]. A study investigating lipid metabolism in PDAC uncovered a link between oncogenic KRAS mutation and increased storage of fatty acids in intracellular lipid droplets. The hormone-sensitive lipase (HSL) was identified as a key player, with its suppression by oncogenic KRAS contributing to tumor cell invasion [88]. Achieving high RAS activity and the loss of tumor suppressors are critical for PDAC formation [89].

### 5.2. KRAS Mutations on Tumor Development and Progression

**Figure 3:**
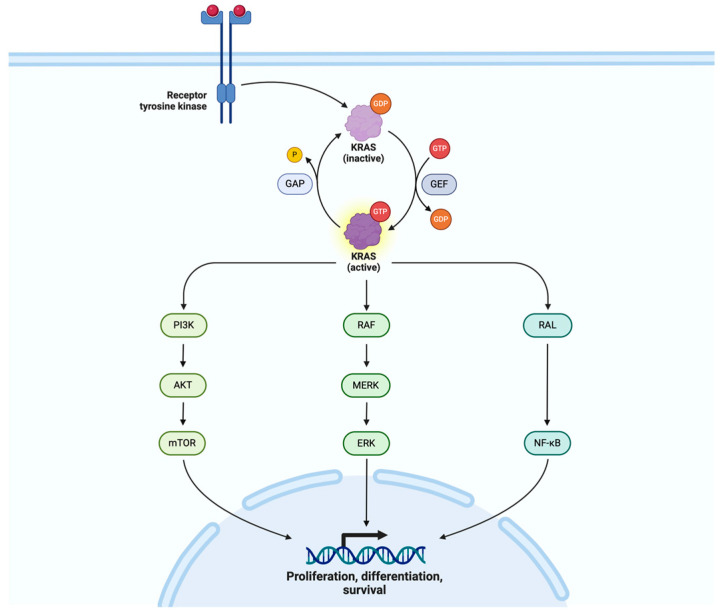
Diagrammatic presentation of the KRAS pathway: The KRAS pathway, including PI3K, RAF and RAL, operates synergistically to regulate key cellular processes such as proliferation, survival and metabolism. Upon activation by extracellular signals, KRAS initiates a signaling cascade that involves the activation of PI3K, RAF and RAL proteins. These downstream effectors act in concert to propagate signaling, leading to the activation of various pathways involved in cell growth and survival. Dysregulation of this synergistic network, often through mutations in KRAS or its effectors, contributes to cancer development and progression. Created with BioRender.com (accessed on 27 February 2024).

In pancreatic cancer, deregulated signaling networks contribute to disease progression. The EGFR-KRAS network in Figure 3, with frequent KRAS mutations, activates downstream pathways like RAS-MAPK and PI3K-AKT, promoting cell survival and proliferation. The dysregulation of Hippo signaling, often via YAP/TAZ amplification, plays a crucial role in PDAC initiation and progression, providing a potential therapeutic target. Inflammation, driven by KRAS signaling and proinflammatory cytokines, accelerates PDAC development, impacting cell survival, proliferation and metastasis [37]. The prognostic significance of KRAS mutational status in unresectable pancreatic cancer was explored in a study analyzing plasma DNA samples from 91 patients. KRAS mutations correlated significantly with tumor staging and liver metastases. Patients with plasma KRAS mutations exhibited significantly shorter median survival times [98]. Berrozpe et al. revealed that 71% of pancreatic cancer cases demonstrated mutations in the *KRAS* gene, primarily at codon 12, indicative of the pivotal role of these mutations in pancreatic tumorigenesis [123]. A multicentric study of advanced PDAC patients found that the G12D KRAS mutation was significantly associated with worse overall survival. This association held true, even in the subgroup that received chemotherapy, highlighting the independent negative prognostic impact of the G12D KRAS mutation in unresectable pancreatic cancer [99]. KRAS mutations, particularly the G12D variant, were associated with reduced median survival time. The combination of KRAS and CDKN2A mutational status was proposed as a potential independent prognostic marker [52]. Hayashi et al. revealed that KRAS has been the most frequently mutated gene, identified in 96% of cases. While mutations in CDKN2A, TP53 and SMAD4 were also prevalent, occurring in 7–42% of cases, nonsynonymous mutations in other cancer-related genes were rare. Survival analyses demonstrated that the number of mutated driver genes, rather than mutations in individual genes, was significantly associated with overall survival. Patients with 0 to 2 mutated genes had a substantially longer median overall survival compared to those with 3 mutated genes [100].

Mouse models were employed to investigate the cooperative impact of p16 inactivation and KRAS activation on pancreatic tumor development. The results demonstrated that while p16 inactivation alone did not initiate tumorigenesis, combined with KRAS activation, it significantly accelerated tumor progression and metastasis, closely resembling human pancreatic ductal adenocarcinoma. Loss of the wild-type KRAS allele further promoted tumor cell proliferation and metastasis [90]. In examining localized pancreatic and ampullary adenocarcinomas, it was found that KRAS mutations were present in 80% of PDAC and 67% of ampullary adenocarcinomas. Notably, KRAS mutations served as an independent prognostic biomarker for shorter overall survival in chemotherapy-naive patients with ampullary adenocarcinoma [101]. A meta-analysis delved into KRAS mutations in pancreatic preneoplastic lesions associated with PDAC and chronic pancreatitis (CP). KRAS mutations were found in 10% of CP lesions and 44% of PDAC lesions, with an increase in frequency with the progression of PanIN grade [102].

### 5.3. Targeted Therapies for KRAS-Mutant Pancreatic Cancer

In the pursuit of targeted therapies for KRAS-mutant pancreatic cancer, diverse strategies have emerged, each being promising in addressing the challenges posed by this notoriously resistant oncogene. One innovative approach employs a CRISPR-Cas13a system for precise targeting of mutant KRAS at the transcriptional level [124]. Utilizing Leptotrichia-derived Cas13a proteins and optimized crRNAs demonstrated robust knockdown of KRAS-G12D mRNA, inhibiting downstream signaling pathways and impeding tumor growth in vivo [91]. Moreover, KRAS-independent survival was observed in some PDAC cells, with the PI3K pathway playing a compensatory role. The study proposed combined KRAS and PI3K inhibition as a therapeutic strategy for PDAC [92]. The potential therapeutic implications involve targeting both MEK and PI3K pathways in pancreatic cancer treatment [93]. Efforts to develop therapeutics targeting mutant KRAS have predominantly focused on inhibiting KRAS effector signaling pathways, with a key emphasis on the RAF-MEK-ERK cascade [107]. Additionally, KRAS WT cases showed higher microsatellite instability and tumor mutational burden, potentially making them more responsive to immune checkpoint inhibitor therapies [103]. Combinatorial approaches targeting multiple KRAS effector pathways, such as MAP kinase and PI3K/AKT, are considered, along with therapies addressing the adaptability of metabolic pathways and the heterogeneity of pancreatic tumors.

Expanding on KRAS mutations, a study utilizing EUS-FNA tissue samples and ctDNA unveiled that KRAS as mutations were detected in 74.7% of EUS-FNA samples and 62.6% of ctDNA samples. The G12V and G12D mutations were prevalent, and their presence in ctDNA was associated with significantly shorter median survival times [104]. In the context of metastatic PDAC, detectable ctDNA has been associated with clinical outcomes, and ctDNA dynamics provided more significant prognostic information compared to the standard biomarker CA19-9 [105]. SiRNA-based approaches also emerge as promising therapeutic strategies. One study employs siRNA vectors targeting specific KRAS mutations, revealing their specificity in inhibiting both wild-type and mutant forms of KRAS, consequently inhibiting cell proliferation and viability [94]. Silencing mutant KRAS in human pancreatic cancer cells using specific siRNA effectively reduces KRAS expression, leading to a dose-dependent decrease in KRAS protein levels. This results in significant reductions in cell proliferation, colony formation and altered cell cycle proteins, indicating cell-cycle arrest. Apoptosis is observed, accompanied by reduced cell migration, altered angiogenic factors and changes in glucose metabolism [125]. Another investigation delves into the design of a biodegradable matrix, the LODER, containing anti-KRASG12D siRNA for sustained local delivery. This approach effectively inhibited pancreatic cancer cell growth in vitro and demonstrated tumor growth inhibition in xenograft and syngeneic mouse models [95]. Furthermore, in a clinical trial involving siG12D-LODER, a sustained release siRNA matrix, in combination with chemotherapy, promising outcomes were observed, including inhibited tumor growth, reduced metastasis development and improved survival rates in patients with locally advanced PDAC [106]. Expanding the therapeutic arsenal, exosomes have been explored as carriers for targeted delivery of siRNA against oncogenic KRAS. Exosomes expressing CD47 demonstrated enhanced circulation and accumulation in pancreatic tumors, effectively reducing KrasG12D mRNA levels and inhibiting tumor growth in mouse models [96]. Additionally, targeting deregulated metabolic pathways in PDAC has been investigated, with a focus on disrupting non-canonical redox homeostasis through GLS-1 inhibition and blocking nutrient salvage pathways [108].

## 6. Conclusions

In summary, the molecular profiling of pancreatic tumors has unveiled critical insights into the genomic landscape, revealing the prominence of key players such as TP53, CDKN2A, SMAD4 and KRAS. These findings underscore the heterogeneity and complexity of pancreatic cancer, emphasizing the need for tailored therapeutic strategies. The potential for personalized approaches, guided by individual tumor molecular profiles, stands as a beacon of hope for improving treatment outcomes. The prognostic significance of specific molecular signatures in predicting patient outcomes offers promise but requires further exploration.

## Figures and Tables

**Table 1:** This table summarizes key cancer-related genes, their chromosomal locations and their roles in nonclinical and clinical studies. TP53, CDKN2A and SMAD4 are tumor suppressor genes, while KRAS is an oncogene. The evidence from both nonclinical and clinical studies supports their respective roles in cancer progression.

Gene	Function	Chromosomal Location	Nonclinical Studies	Clinical Studies	Review
***TP53***	Tumor suppressor gene	17p13.1	[17,18,19,20]	[21,22,23,24,25,26,27,28,29,30,31,32,33]	[34,35,36,37]
***CDKN2A***	Tumor suppressor gene	9p21.3	[38,39]	[40,41,42,43,44,45,46,47,48,49,50,51,52,53,54,55,56]	[57,58,59,60,61]
***SMAD4***	Tumor suppressor gene	18q21.1	[62,63,64,65]	[66,67,68,69,70,71,72,73,74,75,76,77,78,79,80]	[81,82,83,84,85]
***KRAS***	Oncogene	12p12.1	[86,87,88,89,90,91,92,93,94,95,96]	[97,98,99,100,101,102,103,104,105,106]	[107,108]

## Data Availability

Data used in this study are presented within the manuscript.

## References

[1] Siegel R.L., Miller K.D., Wagle N.S., Jemal A. (2023). Cancer statistics, 2023. CA Cancer J. Clin..

[2] Pathology Outlines. https://www.pathologyoutlines.com/topic/pancreaswho.html.

[3] Maitra A., Hruban R.H. (2008). Pancreatic cancer. Annu. Rev. Pathol..

[4] Saiki Y., Jiang C., Ohmuraya M., Furukawa T. (2021). Genetic Mutations of Pancreatic Cancer and Genetically Engineered Mouse Models. Cancers.

[5] Hu H.-F., Ye Z., Qin Y., Xu X.-W., Yu X.-J., Zhuo Q.-F., Ji S.-R. (2021). Mutations in key driver genes of pancreatic cancer: Molecularly targeted therapies and other clinical implications. Acta Pharmacol. Sin..

[6] Özdoğan M., Papadopoulou E., Tsoulos N., Tsantikidi A., Mariatou V.M., Tsaousis G., Kapeni E., Bourkoula E., Fotiou D., Kapetsis G. (2021). Comprehensive tumor molecular profile analysis in clinical practice. BMC Med. Genom..

[7] Ryan K.M., Phillips A.C., Vousden K.H. (2001). Regulation and function of the p53 tumor suppressor protein. Curr. Opin. Cell Biol..

[8] Sausen M., Phallen J., Adleff V., Jones S., Leary R.J., Barrett M.T., Anagnostou V., Parpart-Li S., Murphy D., Kay Li Q. (2015). Clinical implications of genomic alterations in the tumour and circulation of pancreatic cancer patients. Nat. Commun..

[9] Kim H., Saka B., Knight S., Borges M., Childs E., Klein A., Wolfgang C., Herman J., Adsay V.N., Hruban R.H. (2014). Having pancreatic cancer with tumoral loss of ATM and normal TP53 protein expression is associated with a poorer prognosis. Clin. Cancer Res..

[10] Raimondi S., Maisonneuve P., Lowenfels A.B. (2009). Epidemiology of pancreatic cancer: An overview. Nat. Rev. Gastroenterol. Hepatol..

[11] Caparello C., Meijer L.L., Garajova I., Falcone A., Le Large T.Y., Funel N., Kazemier G., Peters G.J., Vasile E., Giovannetti E. (2016). FOLFIRINOX and translational studies: Towards personalized therapy in pancreatic cancer. World J. Gastroenterol..

[12] Ilic M., Ilic I. (2016). Epidemiology of pancreatic cancer. World J. Gastroenterol..

[13] Rawla P., Sunkara T., Gaduputi V. (2019). Epidemiology of Pancreatic Cancer: Global Trends, Etiology and Risk Factors. World J. Oncol..

[14] Hu J.X., Zhao C.F., Chen W.B., Liu Q.C., Li Q.W., Lin Y.Y., Gao F. (2021). Pancreatic cancer: A review of epidemiology, trend, and risk factors. World J. Gastroenterol..

[15] Zhang Q., Zeng L., Chen Y., Lian G., Qian C., Chen S., Li J., Huang K. (2016). Pancreatic Cancer Epidemiology, Detection, and Management. Gastroenterol. Res. Pract..

[16] Huang T.-c.J., Kar S., Javle M. (2010). Personalized therapy for pancreatic cancer: Myth or reality in 2010?. J. Gastrointest. Oncol..

[17] Mello S.S., Flowers B.M., Mazur P.K., Lee J.J., Müller F., Denny S.K., Ferreira S., Hanson K., Kim S.K., Greenleaf W.J. (2023). Multifaceted role for p53 in pancreatic cancer suppression. Proc. Natl. Acad. Sci. USA.

[18] Mello S.S., Valente L.J., Raj N., Seoane J.A., Flowers B.M., McClendon J., Bieging-Rolett K.T., Lee J., Ivanochko D., Kozak M.M. (2017). A p53 Super-tumor Suppressor Reveals a Tumor Suppressive p53-Ptpn14-Yap Axis in Pancreatic Cancer. Cancer Cell.

[19] Morton J.P., Timpson P., Karim S.A., Ridgway R.A., Athineos D., Doyle B., Jamieson N.B., Oien K.A., Lowy A.M., Brunton V.G. (2010). Mutant p53 drives metastasis and overcomes growth arrest/senescence in pancreatic cancer. Proc. Natl. Acad. Sci. USA.

[20] Deer E.L., González-Hernández J., Coursen J.D., Shea J.E., Ngatia J., Scaife C.L., Firpo M.A., Mulvihill S.J. (2010). Phenotype and genotype of pancreatic cancer cell lines. Pancreas.

[21] Zhang F., Zhong W., Li H., Huang K., Yu M., Liu Y. (2021). TP53 Mutational Status-Based Genomic Signature for Prognosis and Predicting Therapeutic Response in Pancreatic Cancer. Front. Cell Dev. Biol..

[22] Liu Y., Cheng L., Song X., Li C., Zhang J., Wang L. (2022). A TP53-associated immune prognostic signature for the prediction of the overall survival and therapeutic responses in pancreatic cancer. Math. Biosci. Eng..

[23] Liu X., Chen B., Chen J., Sun S. (2021). A novel tp53-associated nomogram to predict the overall survival in patients with pancreatic cancer. BMC Cancer.

[24] Gu Y., Ji Y., Jiang H., Qiu G. (2020). Clinical Effect of Driver Mutations of KRAS, CDKN2A/P16, TP53, and SMAD4 in Pancreatic Cancer: A Meta-Analysis. Genet. Test. Mol. Biomark..

[25] Wang Q., Ni Q., Wang X., Zhu H., Wang Z., Huang J. (2015). High expression of RAB27A and TP53 in pancreatic cancer predicts poor survival. Med. Oncol..

[26] Yachida S., White C.M., Naito Y., Zhong Y., Brosnan J.A., Macgregor-Das A.M., Morgan R.A., Saunders T., Laheru D.A., Herman J.M. (2012). Clinical significance of the genetic landscape of pancreatic cancer and implications for identification of potential long-term survivors. Clin. Cancer Res..

[27] Kanda M., Sadakari Y., Borges M., Topazian M., Farrell J., Syngal S., Lee J., Kamel I., Lennon A.M., Knight S. (2013). Mutant TP53 in duodenal samples of pancreatic juice from patients with pancreatic cancer or high-grade dysplasia. Clin. Gastroenterol. Hepatol..

[28] Voutsadakis I.A., Digklia A. (2023). Pancreatic adenocarcinomas without KRAS, TP53, CDKN2A and SMAD4 mutations and CDKN2A/CDKN2B copy number alterations: A review of the genomic landscape to unveil therapeutic avenues. Chin. Clin. Oncol..

[29] McCubrey J.A., Meher A.K., Akula S.M., Abrams S.L., Steelman L.S., LaHair M.M., Franklin R.A., Martelli A.M., Ratti S., Cocco L. (2022). Wild type and gain of function mutant TP53 can regulate the sensitivity of pancreatic cancer cells to chemotherapeutic drugs, EGFR/Ras/Raf/MEK, and PI3K/mTORC1/GSK-3 pathway inhibitors, nutraceuticals and alter metabolic properties. Aging.

[30] Yang S., Che S.P.Y., Kurywchak P., Tavormina J.L., Gansmo L.B., Correa de Sampaio P., Tachezy M., Bockhorn M., Gebauer F., Haltom A.R. (2017). Detection of mutant KRAS and TP53 DNA in circulating exosomes from healthy individuals and patients with pancreatic cancer. Cancer Biol. Ther..

[31] Bloomston M., Frankel W.L., Petrocca F., Volinia S., Alder H., Hagan J.P., Liu C.G., Bhatt D., Taccioli C., Croce C.M. (2007). MicroRNA expression patterns to differentiate pancreatic adenocarcinoma from normal pancreas and chronic pancreatitis. JAMA.

[32] Hashimoto S., Furukawa S., Hashimoto A., Tsutaho A., Fukao A., Sakamura Y., Parajuli G., Onodera Y., Otsuka Y., Handa H. (2019). ARF6 and AMAP1 are major targets of KRAS and TP53 mutations to promote invasion, PD-L1 dynamics, and immune evasion of pancreatic cancer. Proc. Natl. Acad. Sci. USA.

[33] Abrams S.L., Lertpiriyapong K., Yang L.V., Martelli A.M., Cocco L., Ratti S., Falasca M., Murata R.M., Rosalen P.L., Lombardi P. (2018). Introduction of WT-TP53 into pancreatic cancer cells alters sensitivity to chemotherapeutic drugs, targeted therapeutics and nutraceuticals. Adv. Biol. Regul..

[34] McCubrey J.A., Yang L.V., Abrams S.L., Steelman L.S., Follo M.Y., Cocco L., Ratti S., Martelli A.M., Augello G., Cervello M. (2022). Effects of TP53 Mutations and miRs on Immune Responses in the Tumor Microenvironment Important in Pancreatic Cancer Progression. Cells.

[35] Wang S., Zheng Y., Yang F., Zhu L., Zhu X.-Q., Wang Z.-F., Wu X.-L., Zhou C.-H., Yan J.-Y., Hu B.-Y. (2021). The molecular biology of pancreatic adenocarcinoma: Translational challenges and clinical perspectives. Signal Transduct. Target. Ther..

[36] Voutsadakis I.A. (2021). Mutations of p53 associated with pancreatic cancer and therapeutic implications. Ann. Hepatobiliary Pancreat. Surg..

[37] Grant T.J., Hua K., Singh A. (2016). Molecular Pathogenesis of Pancreatic Cancer. Prog. Mol. Biol. Transl. Sci..

[38] Liang J., Fan J., Wang M., Niu Z., Zhang Z., Yuan L., Tai Y., Chen Z., Song S., Wang X. (2018). CDKN2A inhibits formation of homotypic cell-in-cell structures. Oncogenesis.

[39] Tu Q., Hao J., Zhou X., Yan L., Dai H., Sun B., Yang D., An S., Lv L., Jiao B. (2018). CDKN2B deletion is essential for pancreatic cancer development instead of unmeaningful co-deletion due to juxtaposition to CDKN2A. Oncogene.

[40] McWilliams R.R., Wieben E.D., Rabe K.G., Pedersen K.S., Wu Y., Sicotte H., Petersen G.M. (2011). Prevalence of CDKN2A mutations in pancreatic cancer patients: Implications for genetic counseling. Eur. J. Hum. Genet..

[41] Kimura H., Paranal R.M., Nanda N., Wood L.D., Eshleman J.R., Hruban R.H., Goggins M.G., Klein A.P., The Familial Pancreatic Cancer Genome Sequencing P., Roberts N.J. (2022). Functional CDKN2A assay identifies frequent deleterious alleles misclassified as variants of uncertain significance. eLife.

[42] Bartsch D.K., Sina-Frey M., Lang S., Wild A., Gerdes B., Barth P., Kress R., Grützmann R., Colombo-Benkmann M., Ziegler A. (2002). CDKN2A germline mutations in familial pancreatic cancer. Ann. Surg..

[43] Zhen D.B., Rabe K.G., Gallinger S., Syngal S., Schwartz A.G., Goggins M.G., Hruban R.H., Cote M.L., McWilliams R.R., Roberts N.J. (2015). BRCA1, BRCA2, PALB2, and CDKN2A mutations in familial pancreatic cancer: A PACGENE study. Genet. Med..

[44] Jäkel C., Bergmann F., Toth R., Assenov Y., van der Duin D., Strobel O., Hank T., Klöppel G., Dorrell C., Grompe M. (2017). Genome-wide genetic and epigenetic analyses of pancreatic acinar cell carcinomas reveal aberrations in genome stability. Nat. Commun..

[45] Harinck F., Kluijt I., van der Stoep N., Oldenburg R.A., Wagner A., Aalfs C.M., Sijmons R.H., Poley J.W., Kuipers E.J., Fockens P. (2012). Indication for CDKN2A-mutation analysis in familial pancreatic cancer families without melanomas. J. Med. Genet..

[46] Lal G., Liu L., Hogg D., Lassam N.J., Redston M.S., Gallinger S. (2000). Patients with both pancreatic adenocarcinoma and melanoma may harbor germline CDKN2A mutations. Genes Chromosomes Cancer.

[47] Goldstein A.M., Chan M., Harland M., Gillanders E.M., Hayward N.K., Avril M.-F., Azizi E., Bianchi-Scarra G., Bishop D.T., Bressac-de Paillerets B. (2006). High-risk Melanoma Susceptibility Genes and Pancreatic Cancer, Neural System Tumors, and Uveal Melanoma across GenoMEL. Cancer Res..

[48] Goldstein A.M. (2004). Familial melanoma, pancreatic cancer and germline CDKN2A mutations. Hum. Mutat..

[49] Birnbaum D.J., Adélaïde J., Mamessier E., Finetti P., Lagarde A., Monges G., Viret F., Gonçalvès A., Turrini O., Delpero J.-R. (2011). Genome profiling of pancreatic adenocarcinoma. Genes Chromosomes Cancer.

[50] Tang B., Li Y., Qi G., Yuan S., Wang Z., Yu S., Li B., He S. (2015). Clinicopathological Significance of CDKN2A Promoter Hypermethylation Frequency with Pancreatic Cancer. Sci. Rep..

[51] Ibrahim I.S., Vasen H.F.A., Wasser M., Feshtali S., Bonsing B.A., Morreau H., Inderson A., de Vos Tot Nederveen Cappel W.H., van den Hout W.B. (2023). Cost-effectiveness of pancreas surveillance: The CDKN2A-p16-Leiden cohort. United Eur. Gastroenterol. J..

[52] Rachakonda P.S., Bauer A.S., Xie H., Campa D., Rizzato C., Canzian F., Beghelli S., Greenhalf W., Costello E., Schanne M. (2013). Somatic mutations in exocrine pancreatic tumors: Association with patient survival. PLoS ONE.

[53] Masugi Y., Takamatsu M., Tanaka M., Hara K., Inoue Y., Hamada T., Suzuki T., Arita J., Hirose Y., Kawaguchi Y. (2023). Post-operative mortality and recurrence patterns in pancreatic cancer according to KRAS mutation and CDKN2A, p53, and SMAD4 expression. J. Pathol. Clin. Res..

[54] Lin J.-C., Liu T.-P., Yang P.-M. (2020). CDKN2A-Inactivated Pancreatic Ductal Adenocarcinoma Exhibits Therapeutic Sensitivity to Paclitaxel: A Bioinformatics Study. J. Clin. Med..

[55] Doyle A., Kubler M.M., Harris A.C., López A., Govindaraj P., Prins P., Weinberg B.A. (2019). The impact of CDKN2A mutations on overall survival in pancreatic adenocarcinoma. J. Clin. Oncol..

[56] Vaske C.J., Garner C., Seery T.E., Szeto C., Reddy S.K. (2019). Clinical trial screening of CDKN2A genomic alterations in patients with pancreatic cancer and hepatobiliary cancers requires greater precision than somatic sequencing alone. J. Clin. Oncol..

[57] Foulkes W.D., Flanders T.Y., Pollock P.M., Hayward N.K. (1997). The CDKN2A (p16) gene and human cancer. Mol. Med..

[58] Zhao R., Choi B.Y., Lee M.H., Bode A.M., Dong Z. (2016). Implications of Genetic and Epigenetic Alterations of CDKN2A (p16(INK4a)) in Cancer. eBioMedicine.

[59] Jiao Y., Feng Y., Wang X. (2018). Regulation of Tumor Suppressor Gene CDKN2A and Encoded p16-INK4a Protein by Covalent Modifications. Biochemistry.

[60] Kimura H., Klein A.P., Hruban R.H., Roberts N.J. (2021). The Role of Inherited Pathogenic CDKN2A Variants in Susceptibility to Pancreatic Cancer. Pancreas.

[61] Wu C., Yang P., Liu B., Tang Y. (2020). Is there a CDKN2A-centric network in pancreatic ductal adenocarcinoma?. OncoTargets Ther..

[62] Liu L., Li Q., Yang L., Li Q., Du X. (2021). SMAD4 Feedback Activates the Canonical TGF-β Family Signaling Pathways. Int. J. Mol. Sci..

[63] Xiong W., He W., Wang T., He S., Xu F., Wang Z., Wang X., Guo H., Ling J., Zhang H. (2022). Smad4 Deficiency Promotes Pancreatic Cancer Immunogenicity by Activating the Cancer-Autonomous DNA-Sensing Signaling Axis. Adv. Sci..

[64] Wang F., Xia X., Yang C., Shen J., Mai J., Kim H.C., Kirui D., Kang Y., Fleming J.B., Koay E.J. (2018). SMAD4 Gene Mutation Renders Pancreatic Cancer Resistance to Radiotherapy through Promotion of Autophagy. Clin. Cancer Res..

[65] Bardeesy N., Cheng K.H., Berger J.H., Chu G.C., Pahler J., Olson P., Hezel A.F., Horner J., Lauwers G.Y., Hanahan D. (2006). Smad4 is dispensable for normal pancreas development yet critical in progression and tumor biology of pancreas cancer. Genes. Dev..

[66] Tascilar M., Skinner H.G., Rosty C., Sohn T., Wilentz R.E., Offerhaus G.J., Adsay V., Abrams R.A., Cameron J.L., Kern S.E. (2001). The SMAD4 protein and prognosis of pancreatic ductal adenocarcinoma. Clin. Cancer Res..

[67] Moore P.S., Sipos B., Orlandini S., Sorio C., Real F.X., Lemoine N.R., Gress T., Bassi C., Klöppel G., Kalthoff H. (2001). Genetic profile of 22 pancreatic carcinoma cell lines. Analysis of K-ras, p53, p16 and DPC4/Smad4. Virchows Arch..

[68] Principe D.R., Underwood P.W., Kumar S., Timbers K.E., Koch R.M., Trevino J.G., Munshi H.G., Rana A. (2022). Loss of SMAD4 Is Associated With Poor Tumor Immunogenicity and Reduced PD-L1 Expression in Pancreatic Cancer. Front. Oncol..

[69] Liang C., Xu J., Meng Q., Zhang B., Liu J., Hua J., Zhang Y., Shi S., Yu X. (2020). TGFB1-induced autophagy affects the pattern of pancreatic cancer progression in distinct ways depending on SMAD4 status. Autophagy.

[70] Wang Z., Li Y., Zhan S., Zhang L., Zhang S., Tang Q., Li M., Tan Z., Liu S., Xing X. (2019). SMAD4 Y353C promotes the progression of PDAC. BMC Cancer.

[71] Fei N., Wen S., Ramanathan R., Hogg M.E., Zureikat A.H., Lotze M.T., Bahary N., Singhi A.D., Zeh H.J., Boone B.A. (2021). SMAD4 loss is associated with response to neoadjuvant chemotherapy plus hydroxychloroquine in patients with pancreatic adenocarcinoma. Clin. Transl. Sci..

[72] Shin S.H., Kim H.J., Hwang D.W., Lee J.H., Song K.B., Jun E., Shim I.K., Hong S.M., Kim H.J., Park K.M. (2017). The DPC4/SMAD4 genetic status determines recurrence patterns and treatment outcomes in resected pancreatic ductal adenocarcinoma: A prospective cohort study. Oncotarget.

[73] Biankin A.V., Morey A.L., Lee C.S., Kench J.G., Biankin S.A., Hook H.C., Head D.R., Hugh T.B., Sutherland R.L., Henshall S.M. (2002). DPC4/Smad4 expression and outcome in pancreatic ductal adenocarcinoma. J. Clin. Oncol..

[74] Shin S.H., Kim S.C., Hong S.M., Kim Y.H., Song K.B., Park K.M., Lee Y.J. (2013). Genetic alterations of K-ras, p53, c-erbB-2, and DPC4 in pancreatic ductal adenocarcinoma and their correlation with patient survival. Pancreas.

[75] Oshima M., Okano K., Muraki S., Haba R., Maeba T., Suzuki Y., Yachida S. (2013). Immunohistochemically detected expression of 3 major genes (CDKN2A/p16, TP53, and SMAD4/DPC4) strongly predicts survival in patients with resectable pancreatic cancer. Ann. Surg..

[76] Ritterhouse L.L., Wu E.Y., Kim W.G., Dillon D.A., Hirsch M.S., Sholl L.M., Agoston A.T., Setia N., Lauwers G.Y., Park D.Y. (2019). Loss of SMAD4 protein expression in gastrointestinal and extra-gastrointestinal carcinomas. Histopathology.

[77] Crane C.H., Varadhachary G.R., Yordy J.S., Staerkel G.A., Javle M.M., Safran H., Haque W., Hobbs B.D., Krishnan S., Fleming J.B. (2011). Phase II trial of cetuximab, gemcitabine, and oxaliplatin followed by chemoradiation with cetuximab for locally advanced (T4) pancreatic adenocarcinoma: Correlation of Smad4(Dpc4) immunostaining with pattern of disease progression. J. Clin. Oncol..

[78] Xing S., Yang H., Liu J., Zheng X., Feng J., Li X., Li W. (2016). Prognostic Value of SMAD4 in Pancreatic Cancer: A Meta-Analysis. Transl. Oncol..

[79] Yamada S., Fujii T., Shimoyama Y., Kanda M., Nakayama G., Sugimoto H., Koike M., Nomoto S., Fujiwara M., Nakao A. (2015). SMAD4 Expression Predicts Local Spread and Treatment Failure in Resected Pancreatic Cancer. Pancreas.

[80] Xu J.Z., Wang W.Q., Zhang W.H., Xu H.X., Gao H.L., Zhang S.R., Wu C.T., Li S., Li H., Xu J. (2019). The Loss of SMAD4/DPC4 Expression Associated with a Strongly Activated Hedgehog Signaling Pathway Predicts Poor Prognosis in Resected Pancreatic Cancer. J. Cancer.

[81] Zhao M., Mishra L., Deng C.X. (2018). The role of TGF-β/SMAD4 signaling in cancer. Int. J. Biol. Sci..

[82] McCarthy A.J., Chetty R. (2018). Smad4/DPC4. J. Clin. Pathol..

[83] Dardare J., Witz A., Merlin J.L., Gilson P., Harlé A. (2020). SMAD4 and the TGFβ Pathway in Patients with Pancreatic Ductal Adenocarcinoma. Int. J. Mol. Sci..

[84] Ahmed S., Bradshaw A.-D., Gera S., Dewan M.Z., Xu R. (2017). The TGF-β/Smad4 Signaling Pathway in Pancreatic Carcinogenesis and Its Clinical Significance. J. Clin. Med..

[85] Qian Y., Gong Y., Fan Z., Luo G., Huang Q., Deng S., Cheng H., Jin K., Ni Q., Yu X. (2020). Molecular alterations and targeted therapy in pancreatic ductal adenocarcinoma. J. Hematol. Oncol..

[86] Ijichi H., Chytil A., Gorska A.E., Aakre M.E., Fujitani Y., Fujitani S., Wright C.V., Moses H.L. (2006). Aggressive pancreatic ductal adenocarcinoma in mice caused by pancreas-specific blockade of transforming growth factor-beta signaling in cooperation with active Kras expression. Genes. Dev..

[87] Bailey P., Chang D.K., Nones K., Johns A.L., Patch A.M., Gingras M.C., Miller D.K., Christ A.N., Bruxner T.J., Quinn M.C. (2016). Genomic analyses identify molecular subtypes of pancreatic cancer. Nature.

[88] Rozeveld C.N., Johnson K.M., Zhang L., Razidlo G.L. (2020). KRAS controls pancreatic cancer cell lipid metabolism and invasive potential through the lipase HSL. Cancer Res..

[89] Ji B., Tsou L., Wang H., Gaiser S., Chang D.Z., Daniluk J., Bi Y., Grote T., Longnecker D.S., Logsdon C.D. (2009). Ras activity levels control the development of pancreatic diseases. Gastroenterology.

[90] Qiu W., Sahin F., Iacobuzio-Donahue C.A., Garcia-Carracedo D., Wang W.M., Kuo C.-Y., Chen D., Arking D.E., Lowy A.M., Hruban R.H. (2011). Disruption of p16 and activation of Kras in pancreas increase ductal adenocarcinoma formation and metastasis in vivo. Oncotarget.

[91] Kazi A., Xiang S., Yang H., Chen L., Kennedy P., Ayaz M., Fletcher S., Cummings C., Lawrence H.R., Beato F. (2019). Dual farnesyl and geranylgeranyl transferase inhibitor thwarts mutant KRAS-driven patient-derived pancreatic tumors. Clin. Cancer Res..

[92] Muzumdar M.D., Chen P.-Y., Dorans K.J., Chung K.M., Bhutkar A., Hong E., Noll E.M., Sprick M.R., Trumpp A., Jacks T. (2017). Survival of pancreatic cancer cells lacking KRAS function. Nat. Commun..

[93] Hofmann I., Weiss A., Elain G., Schwaederle M., Sterker D., Romanet V., Schmelzle T., Lai A., Brachmann S.M., Bentires-Alj M. (2012). K-RAS mutant pancreatic tumors show higher sensitivity to MEK than to PI3K inhibition in vivo. PLoS ONE.

[94] Réjiba S., Wack S., Aprahamian M., Hajri A. (2007). K-ras oncogene silencing strategy reduces tumor growth and enhances gemcitabine chemotherapy efficacy for pancreatic cancer treatment. Cancer Sci..

[95] Zorde Khvalevsky E., Gabai R., Rachmut I.H., Horwitz E., Brunschwig Z., Orbach A., Shemi A., Golan T., Domb A.J., Yavin E. (2013). Mutant KRAS is a druggable target for pancreatic cancer. Proc. Natl. Acad. Sci. USA.

[96] Kamerkar S., LeBleu V.S., Sugimoto H., Yang S., Ruivo C.F., Melo S.A., Lee J.J., Kalluri R. (2017). Exosomes facilitate therapeutic targeting of oncogenic KRAS in pancreatic cancer. Nature.

[97] Amintas S., Fernandez B., Chauvet A., Chiche L., Laurent C., Belleannée G., Marty M., Buscail E., Dabernat S. (2022). KRAS gene mutation quantification in the resection or venous margins of pancreatic ductal adenocarcinoma is not predictive of disease recurrence. Sci. Rep..

[98] Chen H., Tu H., Meng Z., Chen Z., Wang P., Liu L. (2010). K-ras mutational status predicts poor prognosis in unresectable pancreatic cancer. Eur. J. Surg. Oncol. (EJSO).

[99] Bournet B., Muscari F., Buscail C., Assenat E., Barthet M., Hammel P., Selves J., Guimbaud R., Cordelier P., Buscail L. (2016). KRAS G12D mutation subtype is a prognostic factor for advanced pancreatic adenocarcinoma. Clin. Transl. Gastroenterol..

[100] Hayashi H., Kohno T., Ueno H., Hiraoka N., Kondo S., Saito M., Shimada Y., Ichikawa H., Kato M., Shibata T. (2017). Utility of Assessing the Number of Mutated KRAS, CDKN2A, TP53, and SMAD4 Genes Using a Targeted Deep Sequencing Assay as a Prognostic Biomarker for Pancreatic Cancer. Pancreas.

[101] Schultz N.A., Roslind A., Christensen I.J., Horn T., Høgdall E., Pedersen L.N., Kruhøffer M., Burcharth F., Wøjdemann M., Johansen J.S. (2012). Frequencies and prognostic role of KRAS and BRAF mutations in patients with localized pancreatic and ampullary adenocarcinomas. Pancreas.

[102] Löhr M., Klöppel G., Maisonneuve P., Lowenfels A.B., Lüttges J. (2005). Frequency of K-ras mutations in pancreatic intraductal neoplasias associated with pancreatic ductal adenocarcinoma and chronic pancreatitis: A meta-analysis. Neoplasia.

[103] Philip P.A., Azar I., Xiu J., Hall M.J., Hendifar A.E., Lou E., Hwang J.J., Gong J., Feldman R., Ellis M. (2022). Molecular characterization of KRAS wild-type tumors in patients with pancreatic adenocarcinoma. Clin. Cancer Res..

[104] Kinugasa H., Nouso K., Miyahara K., Morimoto Y., Dohi C., Tsutsumi K., Kato H., Matsubara T., Okada H., Yamamoto K. (2015). Detection of K-ras gene mutation by liquid biopsy in patients with pancreatic cancer. Cancer.

[105] Perets R., Greenberg O., Shentzer T., Semenisty V., Epelbaum R., Bick T., Sarji S., Ben-Izhak O., Sabo E., Hershkovitz D. (2018). Mutant KRAS circulating tumor DNA is an accurate tool for pancreatic cancer monitoring. Oncologist.

[106] Golan T., Khvalevsky E.Z., Hubert A., Gabai R.M., Hen N., Segal A., Domb A., Harari G., David E.B., Raskin S. (2015). RNAi therapy targeting KRAS in combination with chemotherapy for locally advanced pancreatic cancer patients. Oncotarget.

[107] Waters A.M., Der C.J. (2018). KRAS: The Critical Driver and Therapeutic Target for Pancreatic Cancer. Cold Spring Harb. Perspect. Med..

[108] Mann K.M., Ying H., Juan J., Jenkins N.A., Copeland N.G. (2016). KRAS-related proteins in pancreatic cancer. Pharmacol. Ther..

[109] Chen Z., Guo Y., Zhao D., Zou Q., Yu F., Zhang L., Xu L. (2021). Comprehensive Analysis Revealed that CDKN2A is a Biomarker for Immune Infiltrates in Multiple Cancers. Front. Cell Dev. Biol..

[110] Medline CDKN2A Gene. https://medlineplus.gov/genetics/gene/cdkn2a/#function.

[111] Zhang D., Wang T., Zhou Y., Zhang X. (2023). Comprehensive analyses of cuproptosis-related gene CDKN2A on prognosis and immunologic therapy in human tumors. Medicine.

[112] (2024). CDKN2A Cyclin Dependent Kinase Inhibitor 2A [Homo Sapiens (Human)]. https://www.ncbi.nlm.nih.gov/gene/1029.

[113] MedlinePlus SMAD4 Gene. https://medlineplus.gov/genetics/gene/smad4/.

[114] Du X., Li Q., Yang L., Liu L., Cao Q., Li Q. (2020). SMAD4 activates Wnt signaling pathway to inhibit granulosa cell apoptosis. Cell Death Dis..

[115] Du X., Pan Z., Li Q., Liu H., Li Q. (2018). SMAD4 feedback regulates the canonical TGF-β signaling pathway to control granulosa cell apoptosis. Cell Death Dis..

[116] Miyaki M., Kuroki T. (2003). Role of Smad4 (DPC4) inactivation in human cancer. Biochem. Biophys. Res. Commun..

[117] Norris A.M., Gore A., Balboni A., Young A., Longnecker D.S., Korc M. (2013). AGR2 is a SMAD4-suppressible gene that modulates MUC1 levels and promotes the initiation and progression of pancreatic intraepithelial neoplasia. Oncogene.

[118] Ungefroren H., Konukiewitz B., Braun R., Wellner U.F., Keck T., Marquardt J.U. (2023). Elucidation of the Role of SMAD4 in Epithelial-Mesenchymal Plasticity: Does It Help to Better Understand the Consequences of DPC4 Inactivation in the Malignant Progression of Pancreatic Ductal Adenocarcinoma?. Cancers.

[119] Wan R., Feng J., Tang L. (2021). Consequences of Mutations and Abnormal Expression of SMAD4 in Tumors and T Cells. OncoTargets Ther..

[120] Lovely B. (2023). Explorations Unearth New Potential of KRAS Mutations in Pancreatic Cancer. Oncol. Live.

[121] Beganovic S. (2009). Clinical significance of the KRAS mutation. Bosn. J. Basic. Med. Sci..

[122] Storz P. (2017). KRas, ROS and the initiation of pancreatic cancer. Small GTPases.

[123] Berrozpe G., Schaeffer J., Peinado M.A., Real F.X., Perucho M. (1994). Comparative analysis of mutations in the p53 and K-ras genes in pancreatic cancer. Int. J. Cancer.

[124] Zhao X., Liu L., Lang J., Cheng K., Wang Y., Li X., Shi J., Wang Y., Nie G. (2018). A CRISPR-Cas13a system for efficient and specific therapeutic targeting of mutant KRAS for pancreatic cancer treatment. Cancer Lett..

[125] Fleming J.B., Shen G.-L., Holloway S.E., Davis M., Brekken R.A. (2005). Molecular consequences of silencing mutant K-ras in pancreatic cancer cells: Justification for K-ras–directed therapy. Mol. Cancer Res..

